# 5,6-Dimethyl-1,2,9,10-tetra­hydro­pyrano[3,2-*f*]chromene-3,8-dione

**DOI:** 10.1107/S1600536812027699

**Published:** 2012-06-27

**Authors:** Shailesh K. Goswami, Lyall R. Hanton, C. John McAdam, Stephen C. Moratti, Jim Simpson

**Affiliations:** a Department of Chemistry, University of Otago, PO Box 56, Dunedin, New Zealand

## Abstract

The title mol­ecule, C_14_H_14_O_4_, lies on a twofold rotation axis that bis­ects the central benzene ring, with only one half-mol­ecule in the asymmetric unit. The pyran­one systems adopt distorted twist- boat conformations, with the two methyl­ene C atoms displaced by 0.537 (1) and 0.163 (2) Å from the best-fit plane through the remaining five C and O atoms (r.m.s. deviation = 0.073 Å). In the crystal, bifurcated C—H⋯(O,O) hydrogen bonds link pairs of adjacent mol­ecules in an obverse fashion, stacking mol­ecules along *c*. These contacts are further stabilized by very weak π–π inter­actions between adjacent benzene rings with centroid–centroid distances of 4.1951 (4) Å. Additional C—H⋯O contacts link these stacks, giving a three-dimensional network.

## Related literature

For the synthesis, see: Lecea *et al.* (2010 ▶). For details of the Cambridge Structural Database, see: Allen (2002 ▶) and for related structures, see: Cameron *et al.* (2011 ▶); Goswami *et al.* (2011 ▶). For standard bond lengths, see: Allen *et al.* (1987 ▶).
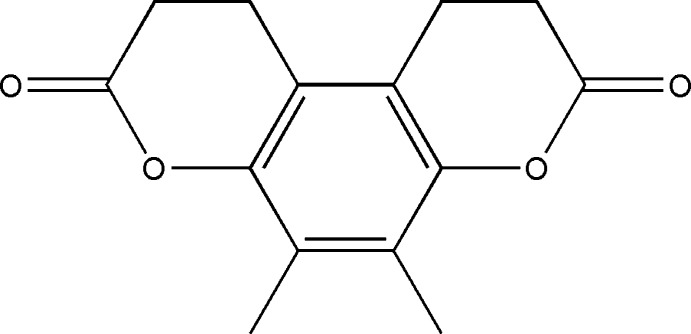


## Experimental

### 

#### Crystal data

C_14_H_14_O_4_
*M* *_r_* = 246.25
Monoclinic, 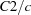

*a* = 16.0726 (2) Å
*b* = 8.7982 (1) Å
*c* = 8.0555 (1) Å
β = 96.1134 (7)°
*V* = 1132.65 (2) Å^3^
*Z* = 4
Mo *K*α radiation
μ = 0.11 mm^−1^
*T* = 92 K
0.53 × 0.50 × 0.22 mm

#### Data collection

Bruker APEXII CCD area-detector diffractometer
Absorption correction: multi-scan (*SADABS*; Bruker, 2011 ▶) *T*_min_ = 0.570, *T*_max_ = 0.748
9744 measured reflections
2989 independent reflections
2358 reflections with *I* > 2σ(*I*)
*R*_int_ = 0.038

#### Refinement

*R*[*F* ^2^ > 2σ(*F* ^2^)] = 0.050
*wR*(*F* ^2^) = 0.148
*S* = 1.08
2989 reflections
83 parameters
H-atom parameters constrained
Δρ_max_ = 0.47 e Å^−3^
Δρ_min_ = −0.38 e Å^−3^

### 

Data collection: *APEX2* (Bruker, 2011 ▶); cell refinement: *APEX2* (Bruker, 2011 ▶) and *SAINT* (Bruker, 2011 ▶); data reduction: *SAINT*; program(s) used to solve structure: *SHELXS97* (Sheldrick, 2008 ▶) and *TITAN2000* (Hunter & Simpson, 1999 ▶); program(s) used to refine structure: *SHELXL97* (Sheldrick, 2008 ▶) and *TITAN2000*; molecular graphics: *SHELXTL* (Sheldrick, 2008 ▶) and *Mercury* (Macrae *et al.*, 2008 ▶); software used to prepare material for publication: *SHELXL97*, *enCIFer* (Allen *et al.*, 2004 ▶), *PLATON* (Spek, 2009 ▶) and *publCIF* (Westrip 2010 ▶).

## Supplementary Material

Crystal structure: contains datablock(s) global, I. DOI: 10.1107/S1600536812027699/bt5941sup1.cif

Structure factors: contains datablock(s) I. DOI: 10.1107/S1600536812027699/bt5941Isup2.hkl

Supplementary material file. DOI: 10.1107/S1600536812027699/bt5941Isup3.cml

Additional supplementary materials:  crystallographic information; 3D view; checkCIF report

## Figures and Tables

**Table 1:** Hydrogen-bond geometry (Å, °)

*D*—H⋯*A*	*D*—H	H⋯*A*	*D*⋯*A*	*D*—H⋯*A*
C61—H61*A*⋯O1^i^	0.98	2.54	3.3907 (11)	145
C2—H2*A*⋯O1^ii^	0.99	2.60	3.4301 (12)	142
C2—H2*A*⋯O2^ii^	0.99	2.64	3.4813 (10)	143
C2—H2*B*⋯O1^iii^	0.99	2.44	3.3528 (11)	154

Symmetry codes: (i) 
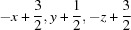
; (ii) 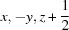; (iii) 
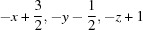
.

**Fig. 1.**
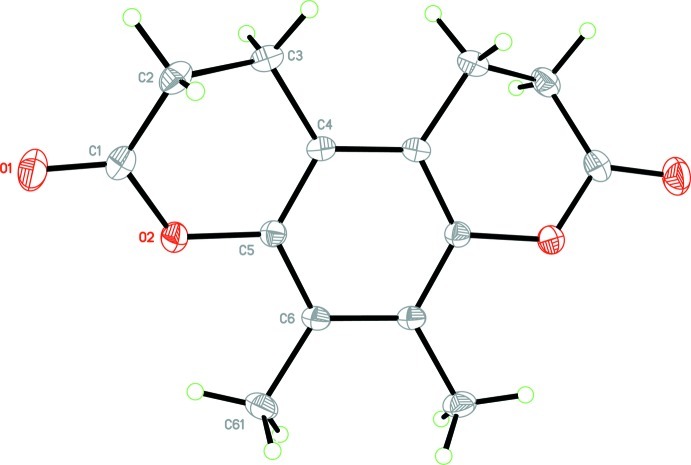
The structure of (I) with ellipsoids drawn at the 50% probability level.

**Fig. 2.**
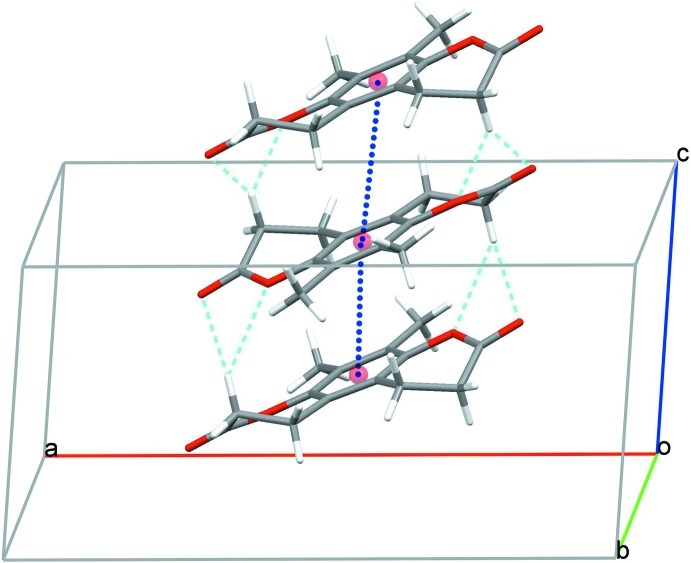
Bifurcated C–H···O hydrogen bonds (dashed lines), augmented by π–π stacking interactions (dotted lines) stacking molecules along c.

**Fig. 3.**
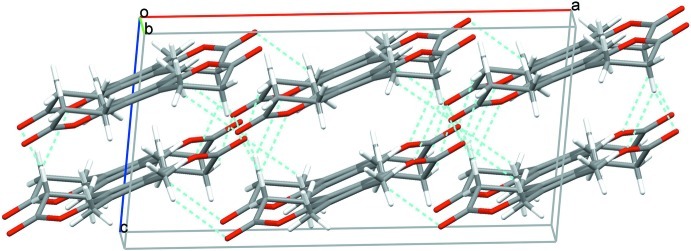
Layers of molecules in planes parallel to (101). Hydrogen bonds are drawn as dashed lines.

**Fig. 4.**
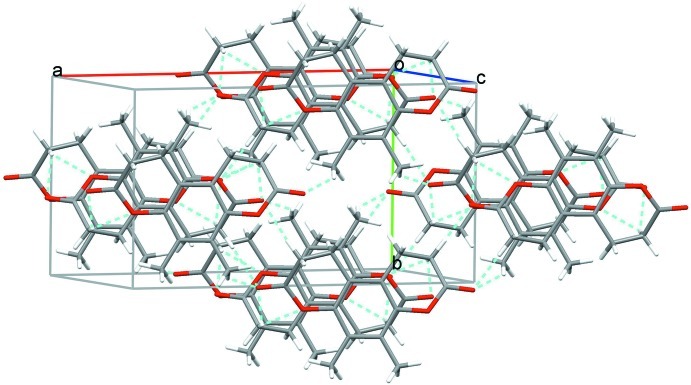
Overall packing of (I) with hydrogen bonds drawn as dashed lines.

## supplementary crystallographic information

### Comment

Our current research interests involve the preparation of redox monomers for the synthesis of electroactive gels. The title compound (I), a chromene-dione was obtained as a by-product of the synthesis of the desired 6-hydroxy-7,8-dimethylchroman-2-one as previously reported by Lecea *et al.* (2010). The chromene-dione (I) provides access to an exciting new redox-active cross-linker in three steps through ring opening, oxidation and condensation reactions.

The asymmetric unit of the title compound contains only one half of the molecule, which lies on a twofold rotation axis that bisects the central benzene ring (Fig 1) The pyranone ring systems adopt distorted twist boat conformations, with the C2 and C3 methylene carbon atoms displaced by 0.537 (1) and 0.163 (2) Å respectively from the best fit plane through C1/(O1)/O2/C5/C4 which has an r.m.s. deviation 0.073 Å. A search of the Cambridge Database (Allen, 2002) revealed no comparable compounds, with or without substitution on the benzene ring. However we have previously reported closely related chroman-2-one derivatives without a second pyranone ring system (Goswami *et al.*, 2011, Cameron *et al.*, 2011). Bond lengths in the structure are not unusual (Allen *et al.*, 1987) and are comparable to those in the chroman-2-one compounds mentioned previously.

In the crystal structure, bifurcated C2–H2A···O1 hydrogen bonds link pairs of adjacent molecules in an obverse fashion stacking molecules along *c*. Very weak π–π interactions, *Cg*···*Cg* = 4.1951 (4) Å, between adjacent benzene rings bolster these contacts further, Fig. 2. Additional C–H···O contacts, Table 1, generate layers of molecules in planes parallel to (1,0,1), Fig 3 while the overall result of this series of contacts is an extended three dimensional network, Fig. 4.

### Experimental

The title compound was obtained as a by-product from a Friedel-Crafts type addition reaction of 2,3-dimethylhydroquinone with acrylic acid during the synthesis of 6-hydroxy-7,8-dimethylchroman-2-one (Lecea *et al.*, 2010). Following work-up according to the literature, X-ray quality crystals of (I) were obtained from dichloromethane solution.

### Refinement

All H-atoms bound to carbon were refined using a riding model with d(C—H) = 0.99 Å, *U*_iso_=1.2*U*_eq_ (C) for methylene and 0.98 Å, *U*_iso_ = 1.5*U*_eq_ (C) for CH_3_ H atoms.

### Crystal data

**Table:**

C_14_H_14_O_4_	*F*(000) = 520
*M**_r_* = 246.25	*D*_x_ = 1.444 Mg m^−^^3^
Monoclinic, *C*2/*c*	Mo *K*α radiation, λ = 0.71073 Å
Hall symbol: -C 2yc	Cell parameters from 3955 reflections
*a* = 16.0726 (2) Å	θ = 2.6–38.3°
*b* = 8.7982 (1) Å	µ = 0.11 mm^−^^1^
*c* = 8.0555 (1) Å	*T* = 92 K
β = 96.1134 (7)°	Rectangular block, yellow
*V* = 1132.65 (2) Å^3^	0.53 × 0.50 × 0.22 mm
*Z* = 4	

### Data collection

**Table:**

Bruker APEXII CCD area-detector diffractometer	2989 independent reflections
Radiation source: fine-focus sealed tube	2358 reflections with *I* > 2σ(*I*)
Graphite monochromator	*R*_int_ = 0.038
φ & ω scans	θ_max_ = 39.3°, θ_min_ = 3.6°
Absorption correction: multi-scan (*SADABS*; Bruker, 2011)	*h* = −27→26
*T*_min_ = 0.570, *T*_max_ = 0.748	*k* = −14→7
9744 measured reflections	*l* = −13→14

### Refinement

**Table:**

Refinement on *F*^2^	Primary atom site location: structure-invariant direct methods
Least-squares matrix: full	Secondary atom site location: difference Fourier map
*R*[*F*^2^ > 2σ(*F*^2^)] = 0.050	Hydrogen site location: inferred from neighbouring sites
*wR*(*F*^2^) = 0.148	H-atom parameters constrained
*S* = 1.08	*w* = 1/[σ^2^(*F*_o_^2^) + (0.0702*P*)^2^ + 0.4368*P*] where *P* = (*F*_o_^2^ + 2*F*_c_^2^)/3
2989 reflections	(Δ/σ)_max_ < 0.001
83 parameters	Δρ_max_ = 0.47 e Å^−^^3^
0 restraints	Δρ_min_ = −0.38 e Å^−^^3^

### Special details

**Table:**

Geometry. All e.s.d.'s (except the e.s.d. in the dihedral angle between two l.s. planes) are estimated using the full covariance matrix. The cell e.s.d.'s are taken into account individually in the estimation of e.s.d.'s in distances, angles and torsion angles; correlations between e.s.d.'s in cell parameters are only used when they are defined by crystal symmetry. An approximate (isotropic) treatment of cell e.s.d.'s is used for estimating e.s.d.'s involving l.s. planes.
Refinement. Refinement of *F*^2^ against ALL reflections. The weighted *R*-factor *wR* and goodness of fit *S* are based on *F*^2^, conventional *R*-factors *R* are based on *F*, with *F* set to zero for negative *F*^2^. The threshold expression of *F*^2^ > σ(*F*^2^) is used only for calculating *R*-factors(gt) *etc*. and is not relevant to the choice of reflections for refinement. *R*-factors based on *F*^2^ are statistically about twice as large as those based on *F*, and *R*- factors based on ALL data will be even larger.

### Fractional atomic coordinates and isotropic or equivalent isotropic displacement parameters (Å^2^)

**Table:**

	*x*	*y*	*z*	*U*_iso_*/*U*_eq_	
O1	0.76307 (4)	−0.03359 (9)	0.53742 (9)	0.02826 (17)	
C1	0.70230 (5)	−0.04789 (10)	0.61199 (10)	0.01983 (16)	
O2	0.64973 (4)	0.07377 (7)	0.61147 (8)	0.01902 (14)	
C2	0.68037 (5)	−0.18492 (10)	0.70770 (10)	0.02081 (16)	
H2A	0.6997	−0.1700	0.8274	0.025*	
H2B	0.7099	−0.2745	0.6682	0.025*	
C3	0.58596 (5)	−0.21578 (9)	0.68795 (10)	0.01919 (15)	
H3A	0.5684	−0.2533	0.5737	0.023*	
H3B	0.5728	−0.2952	0.7681	0.023*	
C4	0.53894 (4)	−0.07275 (8)	0.71898 (9)	0.01485 (14)	
C5	0.57500 (4)	0.06647 (8)	0.68637 (9)	0.01470 (14)	
C6	0.53839 (4)	0.20612 (8)	0.71558 (9)	0.01519 (14)	
C61	0.57956 (5)	0.35308 (10)	0.67541 (12)	0.02167 (17)	
H61A	0.6030	0.4022	0.7792	0.033*	
H61B	0.5380	0.4204	0.6156	0.033*	
H61C	0.6245	0.3325	0.6053	0.033*	

### Atomic displacement parameters (Å^2^)

**Table:**

	*U*^11^	*U*^22^	*U*^33^	*U*^12^	*U*^13^	*U*^23^
O1	0.0216 (3)	0.0340 (4)	0.0307 (3)	0.0048 (2)	0.0097 (2)	0.0013 (3)
C1	0.0175 (3)	0.0226 (4)	0.0194 (3)	0.0039 (2)	0.0021 (2)	−0.0023 (3)
O2	0.0158 (2)	0.0186 (3)	0.0232 (3)	0.00139 (18)	0.0047 (2)	0.0013 (2)
C2	0.0214 (3)	0.0204 (4)	0.0207 (3)	0.0070 (3)	0.0026 (3)	0.0000 (3)
C3	0.0220 (3)	0.0143 (3)	0.0212 (3)	0.0028 (2)	0.0018 (2)	−0.0018 (2)
C4	0.0165 (3)	0.0124 (3)	0.0152 (3)	0.0007 (2)	−0.0003 (2)	−0.0005 (2)
C5	0.0140 (3)	0.0145 (3)	0.0154 (3)	0.0003 (2)	0.0009 (2)	0.0001 (2)
C6	0.0150 (3)	0.0122 (3)	0.0178 (3)	−0.0004 (2)	−0.0007 (2)	0.0006 (2)
C61	0.0196 (3)	0.0149 (3)	0.0302 (4)	−0.0028 (2)	0.0012 (3)	0.0024 (3)

### Geometric parameters (Å, º)

**Table:**

O1—C1	1.2065 (10)	C3—H3B	0.9900
C1—O2	1.3634 (10)	C4—C5	1.3920 (10)
C1—C2	1.4934 (13)	C4—C4^i^	1.3962 (14)
O2—C5	1.4018 (9)	C5—C6	1.3931 (10)
C2—C3	1.5329 (11)	C6—C6^i^	1.4058 (14)
C2—H2A	0.9900	C6—C61	1.5033 (11)
C2—H2B	0.9900	C61—H61A	0.9800
C3—C4	1.5025 (11)	C61—H61B	0.9800
C3—H3A	0.9900	C61—H61C	0.9800
			
O1—C1—O2	116.80 (8)	C5—C4—C4^i^	118.34 (4)
O1—C1—C2	126.11 (8)	C5—C4—C3	118.59 (7)
O2—C1—C2	117.07 (7)	C4^i^—C4—C3	123.06 (4)
C1—O2—C5	121.45 (6)	C4—C5—C6	123.53 (7)
C1—C2—C3	112.01 (7)	C4—C5—O2	120.97 (6)
C1—C2—H2A	109.2	C6—C5—O2	115.40 (6)
C3—C2—H2A	109.2	C5—C6—C6^i^	118.10 (4)
C1—C2—H2B	109.2	C5—C6—C61	121.25 (7)
C3—C2—H2B	109.2	C6^i^—C6—C61	120.66 (4)
H2A—C2—H2B	107.9	C6—C61—H61A	109.5
C4—C3—C2	110.14 (7)	C6—C61—H61B	109.5
C4—C3—H3A	109.6	H61A—C61—H61B	109.5
C2—C3—H3A	109.6	C6—C61—H61C	109.5
C4—C3—H3B	109.6	H61A—C61—H61C	109.5
C2—C3—H3B	109.6	H61B—C61—H61C	109.5
H3A—C3—H3B	108.1		
			
O1—C1—O2—C5	176.44 (7)	C4^i^—C4—C5—O2	−174.81 (8)
C2—C1—O2—C5	−4.87 (11)	C3—C4—C5—O2	5.95 (11)
O1—C1—C2—C3	−142.07 (9)	C1—O2—C5—C4	−19.48 (11)
O2—C1—C2—C3	39.38 (10)	C1—O2—C5—C6	164.02 (7)
C1—C2—C3—C4	−49.20 (9)	C4—C5—C6—C6^i^	1.15 (14)
C2—C3—C4—C5	27.91 (10)	O2—C5—C6—C6^i^	177.55 (8)
C2—C3—C4—C4^i^	−151.29 (9)	C4—C5—C6—C61	−179.02 (7)
C4^i^—C4—C5—C6	1.40 (14)	O2—C5—C6—C61	−2.62 (11)
C3—C4—C5—C6	−177.84 (7)		

Symmetry code: (i) −*x*+1, *y*, −*z*+3/2.

### Hydrogen-bond geometry (Å, º)

**Table:**

*D*—H···*A*	*D*—H	H···*A*	*D*···*A*	*D*—H···*A*
C61—H61*A*···O1^ii^	0.98	2.54	3.3907 (11)	145
C2—H2*A*···O1^iii^	0.99	2.60	3.4301 (12)	142
C2—H2*A*···O2^iii^	0.99	2.64	3.4813 (10)	143
C2—H2*B*···O1^iv^	0.99	2.44	3.3528 (11)	154

Symmetry codes: (ii) −*x*+3/2, *y*+1/2, −*z*+3/2; (iii) *x*, −*y*, *z*+1/2; (iv) −*x*+3/2, −*y*−1/2, −*z*+1.
